# ‘Idealized’ State 4 and State 3 in Mitochondria vs. Rest and Work in Skeletal Muscle

**DOI:** 10.1371/journal.pone.0117145

**Published:** 2015-02-03

**Authors:** Bernard Korzeniewski

**Affiliations:** Faculty of Biochemistry, Biophysics and Biotechnology, Jagiellonian University, Kraków, Poland

## Abstract

A computer model of oxidative phosphorylation (OXPHOS) in skeletal muscle is used to compare state 3, intermediate state and state 4 in mitochondria with rest and work in skeletal muscle. ‘Idealized’ state 4 and 3 in relation to various ‘experimental’ states 4 and 3 are defined. Theoretical simulations show, in accordance with experimental data, that oxygen consumption (V’O_2_), ADP and P_i_ are higher, while ATP/ADP and Δp are lower in rest than in state 4, because of the presence of basal ATP consuming reactions in the former. It is postulated that moderate and intensive work in skeletal muscle is very different from state 3 in isolated mitochondria. V’O_2_, ATP/ADP, Δp and the control of ATP usage over V’O_2_ are much higher, while ADP and Pi are much lower in the former. The slope of the phenomenological V’O_2_-ADP relationship is much steeper during the rest-work transition than during the state 4-state 3 transition. The work state in intact muscle is much more similar to intermediate state than to state 3 in isolated mitochondria in terms of ADP, ATP/ADP, Δp and metabolic control pattern, but not in terms of V’O_2_. The huge differences between intact muscle and isolated mitochondria are proposed to be caused by the presence of the each-step activation (ESA) mechanism of the regulation of OXPHOS in intact skeletal muscle. Generally, the present study suggests that isolated mitochondria (at least in the absence of Ca^2+^) cannot serve as a good model of OXPHOS regulation in intact skeletal muscle.

## Introduction

State 3 in isolated mitochondria was originally defined by Chance and Williams [1,2] as a state with high external (extramitochondrial) ADP, low external ATP/ADP ratio and high (maximal in isolated mitochondria without Ca^2+^) oxygen consumption (V’O_2_) and ATP synthesis (vATP_s_). State 4, on the other hand, was defined as a state with a very high ATP/ADP ratio, very low ADP, no ATP synthesis and V’O_2_ corresponding exclusively to proton leak. Originally, state 3 was set by an addition of external ADP. After some time most ADP was transformed by oxidative phosphorylation (OXPHOS) in mitochondria into ATP and the system passed to state 4. There is essentially no real steady-state in this kind of experiments, because ATP/ADP changes continuously as ADP is continuously converted to ATP. State 4 and state 3 were set in many other studies by adding to mitochondria suspension appropriate amounts of ATP and ADP.

In several experiments [3–6] an artificial ADP regeneration system (hexokinase + glucose) was used to fix different ADP, ATP/ADP, V’O_2_ and vATP_s_ levels, and thus to establish particular states. At zero hexokinase amount/activity state 4 is present, at saturating (for OXPHOS) hexokinase amount/activity state 3 is reached, and at intermediate hexokinase amount/activity we deal with a state that can be called intermediate state, where intermediate ADP, ATP/ADP, V’O_2_ and vATP_s_ levels are present [3,4]. In such experimental set a quasi steady-state can be reached (approximately constant ATP/ADP for a long time) and the states intermediate between state 4 and state 3 can be studied [3,4]. However, because P_i_ is continuously used in this experimental system (see Discussion), a high P_i_ concentration (usually 10 mM) is added to the mitochondria suspension.

In short, state 4 can be characterized as a state without any ATP usage/production, while state 3 can be characterized as a state with saturating ATP usage/production in the absence of each-step activation (ESA).

However, significantly different ‘experimental’ states 4 and states 3 are present in various experimental studies, even in mitochondria isolated from the same tissue. Brand and Nicholls [7] distinguish state 4 (without oligomycin) and state 4_oligomycin_ (with oligomycin, an inhibitor of ATP synthase that prevents any ATP turnover related to external ATPases possibly present in mitochondria preparation). Additionally, different states 4 can be caused by different quality of mitochondria preparation (fraction of mitochondria with intact, not broken inner membranes) and various respiratory substrates used. Finally, while in isolated mitochondria a high approximately constant P_i_ concentration (most frequently 10 mM) is maintained in all states, in state 4 in intact tissues (e.g., skeletal muscle [8,9]) this concentration is certainly much lower (even lower than in resting muscle, where it equals about 2–3 mM [10–12]).

Similarly, significantly different states 3 can be set by addition of excess of ADP, saturating hexokinase concentration (in the presence of glucose), different respiratory substrates and uncouplers. Also, P_i_ concentration in e.g., intact skeletal muscle during intensive work is much higher than in isolated mitochondria (reaching about 20–30 mM [10–12]). In a theoretical state 3 in intact skeletal muscle (no direct OXPHOS activation by ESA, see below, saturating ATP usage) P_i_ concentration would have to be even higher than at high work.

Therefore, in the present study ‘idealized’ state 4 and state 3 (state 4_id_ and state 3_id_) are defined, of which ‘experimental’ states 4 and 3 are better or worse approximations (see Theoretical Methods and Discussion). Just these ‘idealized’ states are used in computer simulations in the present study.

It is sometimes assumed or postulated, explicitly or implicitly, that in all intact skeletal muscles rest corresponds to state 4, while work corresponds to state 3 or at least is close to state 3 (see e.g., [13,14]). Indeed, under some experimental conditions it seems that electrically-stimulated glycolytic skeletal muscle reaches a state close to state 3 at maximal electrical stimulation (and therefore each-step activation, ESA, is absent, see below) (see e.g., [15]). The linear work-PCr (or work-Cr) dependence observed in the cited study is approximately equivalent to the hyperbolic phenomenological vATPs-ADP relationship (see Figure Five in [16]).

In isolated mitochondria (at least in the absence of Ca^2+^) the only mechanism of the regulation of OXPHOS is the negative feedback through ADP (at nearly constant P_i_). Due to the traditional opinion also in intact skeletal muscle the ATP hydrolysis products, ADP and, to a smaller extent, P_i_, constitute feedback signals that are exclusively responsible for the regulation of OXPHOS. Namely, it is supposed that neural stimulation of a muscle cell causes a release of Ca^2+^ from sarcoplasmic reticulum to cytosol, which activates ATP usage (actomyosin-ATPase and Ca^2+^-ATPase). This causes hydrolysis of ATP to ADP and P_i_ that, in turn, activate OXPHOS. Also several theoretical studies, using computer models, assume that OXPHOS in skeletal muscle [17] and heart [18,19] is regulated exclusively by a negative feedback acting mostly through P_i_ (in the case of skeletal muscle also through ADP). In the discussed computer simulations P_i_ concentration is extremely low (in the micromolar range) in resting skeletal muscle and slowly beating heart, and this concentration increases greatly (by a few orders of magnitude) during rest-to-work or low-to-high-work transition, which contradicts experimental findings (see [20,21] for discussion). However, the authors do not exclude other mechanisms of OXPHOS regulation.

Theoretical studies using a computer model of the skeletal muscle bioenergetic system developed previously by myself and co-workers [22,16,23] and extensively tested by comparison with various experimental data (see e.g., [20,21] for overview) strongly suggested that some cytosolic factor, probably related to cytosolic Ca^2+^ and protein phosphorylation, directly activates all OXPHOS complexes (complex I, complex III, complex IV, ATP synthase, ATP/ADP carrier, P_i_ carrier) in parallel with ATP usage and NADH supply during rest-to-work transition in skeletal muscle (and low-to-high work transition in heart) [22,20,21]). This is the so-called each-step activation (ESA) mechanism. A similar mechanism, called ‘multisite modulation’ was proposed by Fell and Thomas in a more abstract and general way in relation to other pathways, especially glycolysis [25].

This mechanism is absent in isolated mitochondria, at least as long as external Ca^2+^ is not added to mitochondria suspension. It was demonstrated experimentally, using the top-down approach [24,4] to Metabolic Control Analysis (MCA, see ref. [26] for overview) that Ca^2+^ activates both oxidative subsystem (OX: NADH/FADH_2_ supply, complex I, complex III, complex IV) and phosphorylation subsystem (PH: ATP synthase, ATP/ADP carrier, P_i_ carrier) in isolated skeletal muscle mitochondria incubated with sub-saturating concentrations of 2-oxoglutarate, while only PH subsystem was activated with succinate [27]. In a recent work it was demonstrated that Ca^2+^ (in the physiological range) activates about twice essentially all OXPHOS complexes in skeletal muscle mitochondria respiring on glutamate/malate [28]. In brain mitochondria a strong activation of OXPHOS by Ca^2+^ with glutamate/malate as respiratory substrates, a moderate activation with 2-oxoglutarate/malate or isocitrate/malate, and essentially no activation with pyruvate was observed [29]. In heart mitochondria OXPHOS (mostly OX subsystem) is activated with sub-saturating concentration of 2-oxoglutarate, but not with saturating concentration of 2-oxoglutarate or succinate [30].

It was demonstrated that Ca^2+^ activates isolated pyruvate dehydrogenase (PDH), isocitrate dehydrogenase (ICDH) and 2-oxoglutarate dehydrogenase (OGDH) [31,32] as well as aralar (glutamate/aspartate carrier), an element of the malate/aspartate shuttle (MAS) [33,34]. It was also postulated that Ca^2+^ activates ATP synthase in isolated mitochondria [35].

Additionally, unlike in isolated mitochondria, in intact skeletal muscle there is always, also at rest (and in arrested heart), some ATP usage for basal processes that keep the cell alive (protein/RNA synthesis, Na^+^/K^+^ and Ca^2+^ ion circulation). The phenomenological V’O_2_-ADP relationship in different skeletal muscles is much steeper than first order and the slope of this relationship varies dramatically between different muscles (see [21] for review). This was first emphasized by Hochachka [36], who postulated that some (unidentified) enzymes are stimulated by some (unidentified) factor during rest-work transition in skeletal muscle (a ‘latent enzymes hypothesis)’. Generally, one can expect that the kinetic behavior of the bioenergetic system in intact muscle differs significantly from that in isolated mitochondria (at least in the absence of Ca^2+^).

The main purpose of the present research-polemic article is to integrate and explain, using a computer model developed previously, some of the existing experimental data concerning the kinetic behavior of the skeletal muscle energy metabolism system in response to elevated energy demand, and to predict some new system properties. An important element of this task is to explicate and explain the differences between intact skeletal muscle and isolated skeletal muscle mitochondria. ‘Idealized’ state 4 and state 3 (state 3_id_ and state 4_id_) in relation to the plethora of various ‘experimental’ states 4 and states 3 are defined, characterized and used in computer simulations. In particular, the article is intended to support the experimental finding [8] that skeletal muscle at rest is not in state 4 and, first of all, to convince the reader, by referring computer simulations to experimental data, that the working state in intact muscle is very different from state 3 in isolated mitochondria because of the each-step activation mechanism (ESA) acting in the former. The differences in the pattern of metabolic control over V’O_2_ between different states (state 4 _id_, intermediate state, state 3 _id_, rest, work) are discussed. It is intended to further characterize carefully all these states. Generally, the postulated differences in the regulation of OXPHOS between intact skeletal muscle and isolated mitochondria are explicated and discussed. It is shown that the computer model of the skeletal muscle energy metabolism system used, supplemented with ESA, is able to integrate and explain the relevant properties of both the isolated mitochondria and intact skeletal muscle system.

## Methods

Throughout the article I mean by ATP, ADP and P_i_ extramitochondrial (cytosolic in the case of intact cells/tissues) ATP, ADP and P_i_ and not intramitochondrial ATP, ADP and P_i_.

### ‘Idelalized’ state 4 and state 3 vs. ‘experimental’ states 4 and states 3

Very different states 4 and states 3 were set in different experimental studies, even those using mitochondria isolated from the same tissue. Brand and Nicholls [7] distinguished state 4 (in the absence of oligomycin) and state 4_oligomycin_ (in the presence of oligomycin that inhibits ATP synthase and thus prevents any conversion of ADP to ATP by OXPHOS; ADP could be produced by external ATPases possibly present in mitochondria preparations). The authors also emphasize the importance of the quality of mitochondria preparation for the state 4 V’O_2_ and RCR ratio (respiratory control ratio: V’O_2_ in state 3 / V’O_2_ in state 4). Indeed, the procedure of mitochondria preparation is usually a compromise between yield and mitochondria quality (in particular: intactness of their inner membranes). In worse preparations a greater mitochondria fraction has a disrupted inner mitochondria membrane and therefore they are in fact in state 3_unc_ (see below), which significantly elevates their phenomenological ‘state 4’ respiration and lowers their ‘RCR ratio’ in relation to good mitochondria preparations and, especially, to the ‘idealized’ state 4 (see below). In better mitochondria preparations the fraction of mitochondria with preserved integrity of inner membrane is high. State 4 V’O_2_ can be also affected by respiratory substrates used. Finally, P_i_ concentration in isolated mitochondria (usually 10 mM) is much higher than in resting muscle (around 2–3 mM, see [10–12]), which must be still higher than in oligomycin-induced state 4 in intact muscle [8,9].

Similarly, one can distinguish various ‘experimental’ states 3. In isolated mitochondria, this state can be induced by addition of high ADP concentration or by saturating hexokinase amount in the presence of glucose (artificial ADP-regenerating system). V’O_2_ in state 3 depends very significantly on the respiratory substrate used (compare Discussion). It is lowest with pyruvate/malate (NAD related substrates, protons pumped by complexes I, III and IV of the respiratory chain), almost twice higher with succinate (FAD-related substrate, complex I omitted) and several times higher with external reduced cytochrome c or ascorbate+TMPD (complexes I and III omitted, see Discussion). State 3 can be also induced by uncoupler addition (state 3_unc_). In this state ATP synthase, ATP/ADP carrier, P_i_ carrier and possibly ATP usage system are omitted and therefore V’O_2_ is higher than in state 3_ADP_ (high external ADP) or state 3_hex_ (saturating hexokinase amount/activity), because these elements of the system have significant control over V’O_2_ (see e.g., [37]). I am not aware of any experimental method that would allow to obtain state 3 in intact skeletal muscle (no direct activation of OXPHOS by ESA, saturating activity of ATP usage and thus saturating ADP and P_i_ concentrations). However, in such potential (theoretical) state 3 P_i_ concentration would be much higher than in the isolated mitochondria system (usually 10 mM) or even in intact muscle at intensive work (about 20–30 mM [10–12]).

Therefore, I would like to define ‘idealized’ state 4 and state 3 (state 4_id_ and state 3 _id_) that could serve as a reference for ‘experimental’ states 4 and states 3, and of which the ‘experimental’ states 4 and states 3 would be better or worse approximations. In state 4 _id_ 100% of V’O_2_ would be due to proton leak (no ATP production by ATP synthase) and all mitochondria would retain completely intact inner membrane. Mitochondria would respire on possibly ‘physiological’ substrates, mostly pyruvate and/or fatty acids in the case of skeletal muscle. P_i_ concentration would be significantly lower than in isolated mitochondria, but still in the milimollar range (say about 0.5 mM, see below). This would diminish V’O_2_ in relation to the ‘experimental’ state 4 in the isolated mitochondria system with a high (almost) constant P_i_.

In state 3_id_ the direct OXPHOS activation through ESA mechanism would be absent and ATP usage activity would be saturating for OXPHOS. ‘Physiological’ substrates would be used by mitochondria. No inner mitochondria membranes would be disrupted and no uncoupler would be added. P_i_ concentration would be much higher than that usually used in the isolated mitochondria system. This would elevate V’O_2_ in state 3_id_ in relation to the ‘experimental’ state 3_ADP_ (with external ADP added) and state 3_hex_ (with saturating hexokinase amount/activity) in the isolated mitochondria system.

As a result of the above enumerated differences, the ‘idealized’ RCR (RCR_id_, V’O_2_ in state 3_id_ / V’O_2_ in state 4_id_) would be significantly higher than various ‘experimental’ RCRs encountered in experimental studies.

State 4 _id_ and state 3 _id_ were defined in order to reflect possibly well intact cell/tissue conditions, although state 4 and state 3 (the latter at least in most cases) do not appear in vivo under physiological conditions.

Probably the best known (although certainly not perfect) approximation of state 4_id_ in skeletal muscle mitochondria is oligomycin-induced state 4 in intact skeletal muscle [8,9]. In this system all mitochondria retain intact mitochondrial membranes and 100% (presumably) of mitochondrial V’O_2_ in state 4_oligomycin_ is due to proton leak (no ATP synthesis by OXPHOS). Mitochondria respire on ‘physiological’ mixture of substrates and P_i_ in state 4 is certainly much lower than in the isolated mitochondria system. In isolated skeletal muscle mitochondria, state 3 with glutamate/malate and/or fatty acids, with well preserved integrity of inner membrane during mitochondria preparation, and without uncoupler (and perhaps with a very high P_i_) seems to be closest to state 3_id_. I am not aware of any good experimental approximation of state 3_id_ in intact skeletal muscle, perhaps apart from some experimental systems where ESA seems to be for some reasons absent or very low [15]. In my opinion, mostly cytosolic Ca^2+^ is involved in the direct activation of OXPHOS complexes (ESA) during muscle work [22,20,21]. Therefore, for instance ruthenium red (RR, an inhibitor of Ca^2+^ transport into mitochondrial matrix) cannot switch off most of ESA. The control over V’O_2_ is shared between particular OXPHOS complexes and pyruvate carrier in state 3 in skeletal muscle mitochondria, leaving essentially no room for the control exerted by TCA cycle [37]. Therefore, a lack of stimulation by mitochondrial Ca^2+^ of TCA cycle dehydrogenases (pyruvate dehydrogenase, isocitrate degydrogenase, 2-oxoglutarate dehydrogenase) in the presence of RR during muscle work would not affect much the work state V’O_2_ and metabolite concentrations and would not bring the system to state 3.

The ‘idealized’ state 4 and state 3 (state 4_id_ and state 3_id_) will be simulated in the present theoretical study.

It should be clearly emphasized that state 4_id_ and state 3_id_ cannot be reached in 100% in experimental studies. For many purposes trying to approach them would be simply unpractical, contradictory with the aim of an experiment or even counter-productive. However, it seems that they can constitute a reference point for various ‘experimental’ states 4 and states 3 useful in general discussion and for computer modeling.

### Computer model and simulations

The computer model of the skeletal muscle bioenergetic system developed previously [23], based on earlier models of OXPHOS in isolated skeletal muscle mitochondria [38,22] and intact skeletal muscle [16], was used for theoretical studies in the present work. The model takes into account explicitly: particular OXPHOS complexes (complex I, complex III, complex IV, ATP synthase, ATP/ADP carrier, P_i_ carrier), NADH supply, ATP usage, creatine kinase (CK), proton leak, proton efflux/influx to/from blood. It has been broadly validated for different kinetic properties of the system (see e.g., [20,21] for discussion).

Different muscles can have very different values of the maximum VO_2_ and of the slope of the VO_2_-ADP relationship. This can be seen in Figure Nine in a recent work [21], where data from 11 different experiments concerning the VO_2_-ADP relationship in skeletal muscle are collected. However, in all cases the maximum VO_2_ and the slope of the VO_2_-ADP relationship are much (several times) greater than in isolated mitochondria. Therefore, the variability of the bioenergetic characteristics of skeletal muscles does not affect the general conclusions concerning the fundamental difference between intact skeletal muscles and isolated mitochondria. On the other hand, it has been shown in the same paper (Figure Five) that the variability of skeletal muscle properties can be very well explained by different intensities of ESA.

The model used in the present theoretical study is intended to be a model of an ‘average’ or ‘typical’ human skeletal muscle, containing a mixture of oxidative and glycolytic fibers. It assumes that mitochondria occupy, on average, 7% of the cell volume, which is a typical value for human skeletal muscles [39]. In other words, the scaling factor between muscle wet weight and mitochondrial protein content is about 18 g mitochondrial protein per 1 kg of wet muscle weight [40] (assuming that proteins constitute about 25% of mitochondria). I have chosen to express VO_2_ in both intact muscle and isolated mitochondria in ‘intact muscle’ units—mM min^-1^. In the absence of ESA, VO_2_ in intact muscle (wet tissue) expressed in mM O_2_ min^-1^ equals 18 g of mitochondrial protein per 1 kg of wet tissue multiplied by VO_2_ in isolated mitochondria expressed in mmol O_2_ min^-1^ g^-1^ mitochondrial protein. Thus, the value of 3.63 mM min^-1^ calculated in this study for VO_2_ in state 3 in intact muscle (see Table 1) corresponds to 0.202 mmol O_2_ g^-1^ (202 nmol O_2_ mg^-1^) in state 3 in isolated mitochondria.

**Table 1:** Simulated values of V’O_2_, ADP, ATP/ADP, P_i_ and Δp in state 4_id_, state 3 _id_, intermediate state, rest, moderate exercise and intensive exercise.

State (relative k_UT_)	V’O_2_ (mM min^-1^)	ADP (μM)	ATP/ADP	P_i_ (mM)	Δp (mV)
State 4 _id_ (0)	0.19	2.8	2434	0.45	195.4
Intermediate state (15)	1.89	64.3	103	17.4	168.8
State 3 _id_ (31)	3.63	1318	0.9	36.9	153.6
Rest (1)	0.29	6.6	1010	2.7	191.9
Moderate work (30)	3.73	32.3	207	12.2	177.8
Intensive work (80)	9.45	83.6	79	19.1	166.8

V’O_2_ in state 4_id_, intermediate state and state 3 _id_ is scaled for mitochondria in intact skeletal muscle. The phenomenological dependence of V’O_2_ on ADP involves implicitly the dependence on P_i_. The activity of ATP usage (k_UT_ rate constant) is scaled for 1 at rest.

There is quite a big variability of VO_2_ in state 3 in isolated human skeletal muscle mitochondria between different experiments. However, the value simulated in the present study, consistent with the value obtained in [40], is at the upper extreme of this range of variability—compare Table three in [40]. For lower values the difference between isolated mitochondria and intact skeletal muscle would be even larger. Similar VO_2_ in state 3 in isolated human skeletal muscle mitochondria as in [40] were measured in [41] and [42].

In order to compare directly state 4 _id_, intermediate state and state 3 _id_ with rest, moderate work and intensive work states, the model version for intact skeletal muscle was used in all simulations, and V’O_2_ in state 4 _id_, intermediate state and state 3 _id_ was scaled for mitochondria in intact skeletal muscle. This fact does not change the essence of the problem, although state 4 _id_, intermediate state and state 3 _id_ in intact skeletal muscle do not appear in vivo under (at least most) physiological conditions.

The state 4_id_-state 3_id_ transition was simulated by a gradual increase in subsequent simulations of the activity (rate constant k_UT_) of ATP usage (scaled to 1 in resting muscle) from zero (state 4_id_) to the value that is saturating for OXPHOS (state 3_id_). In all simulations steady-state variable values were reached and recorded. There was no direct activation of OXPHOS complexes: their activities (rate constants) remained unchanged. Therefore, OXPHOS was activated only indirectly, through an increase in ADP (and P_i_). This procedure corresponds to a gradual increase in the amount (activity) of an artificial ADP-regenerating system, hexokinase (in the presence of glucose), in experimental studies on isolated mitochondria.

The rest-to-moderate-to-intensive work transition in intact skeletal muscle was simulated by a gradual increase in subsequent simulations of the relative activity (rate constant k_UT_) of ATP usage (scaled to 1 at rest) from 1 (rest) to 30 (moderate work) and further to 80 (intensive work) (in all simulations steady-state variable values were reached and recorded). At the same time, the activities (rate constants) of all OXPHOS complexes and NADH supply were activated n^0.35^ times, where n is the relative value of the rate constant k_UT_ of ATP usage scaled to 1 at rest and the power coefficient of 0.35 corresponds to a moderate ESA intensity (see [21]). This corresponds to the ESA mechanism [22,20,21]. Or, more precisely, this is the so-called mixed mechanism (MM) [21], where all OXPHOS complexes are directly activated (by ESA), but to a smaller extent than ATP usage (e.g., 30^0.35^ = 3.3 times vs. 30 times for moderate exercise or 80^0.35^ = 4.6 times vs. 80 times for intensive exercise). In the result some moderate changes in ADP (and P_i_) take place and the feedback activation mechanism co-operates with ESA mechanism in the regulation of OXPHOS [21]. This differs, in the author’s opinion, skeletal muscle from intact heart in vivo where OXPHOS complexes are directly activated to the same extent as ATP usage (say 5 times) and essentially no changes in intermediate metabolite concentrations (particularly ADP and P_i_) take place (‘pure’ ESA) [21].

Flux control coefficients (FCCs), defined within Metabolic Control Analysis (see [26] for overview), over V’O_2_ for OXPHOS, proton leak and ATP usage were calculated according to the following equation:

$$C_{A_{i}}^{J}=\frac{\partial J/J}{\partial A_{i}/A_{i}}$$ (1)

where dJ/J is a relative change in the flux and dA_i_/A_i_ is a small relative change in a broadly understood enzyme i activity causing the change in the flux. A_i_ can be changed by a change in any relevant parameter (V_m_, k_cat_, E_i_, K_m_, specific inhibitor etc.). FCC can be determined not only for single enzymes, but also for metabolic blocks (like OXPHOS).

FCCs over V’O_2_ flux for OXPHOS, proton leak and ATP usage in particular states were determined by multiplying the rate constants of OXPHOS (all complexes), proton leak or ATP usage by 1.01 (increase by 1%) and recording the new steady-state value of V’O_2_. Therefore, in this case dA_i_/A_i_ = dk_i_/k_i_.The values of FCCs were calculated using Equ. 1.

Similarly, FCCs over V’O_2_ for the oxidation subsystem (OX: NADH supply, complex I, complex III, complex IV), phosphorylation subsystem (PH: ATP usage, ATP synthase, ATP/ADP carrier, P_i_ carrier) and proton leak subsystem (LK) distinguished around Δp (protonmotive force, or, more precisely, ΔΨ: membrane potential) within the top-down approach to Metabolic Control Analysis [24,4] were calculated by increasing the rate constants of the steps belonging to particular subsystems at different ATP usage activities (corresponding to state 4_id_, state 3_id_ and intermediate states) by 1%, recording new steady-state V’O_2_ values and using Equ. 1.

All simulations, figures and tables presented in the present article are original and have been not published before, although some of the ideas and simulations are similar to those presented previously.

The complete model description is available on the web site: http://awe.mol.uj.edu.pl/~benio/

## Results

**Fig 1:**
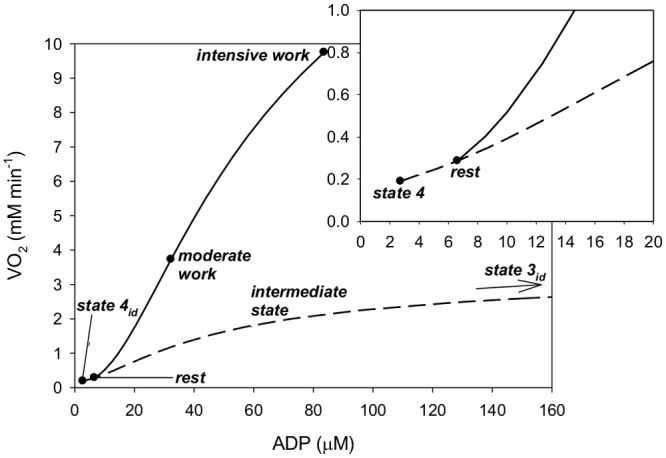
Simulated phenomenological steady-state V’O_2_-ADP relationship in different states. These states comprise: state 4_id_, intermediate state, state 3 _id_, rest, moderate work and intensive work in isolated mitochondria and intact skeletal muscle. Inset: enlarged fragment around state 4 and rest. The presented phenomenological V’O_2_-ADP relationship involves implicitly the V’O_2_-P_i_ relationship. V’O_2_ in isolated mitochondria is scaled for mitochondria in skeletal muscle in order to make a direct comparison.

Computer simulations demonstrate that the rest state in intact skeletal muscle does not correspond exactly to state 4_id_ and, first of all, the work state does not correspond to state 3_id_. This is demonstrated in Fig. 1, which shows the simulated phenomenological dependence of V’O_2_ on ADP during state 4_id_-state 3_id_ transition and during rest-work transition (in both cases the phenomenological V’O_2_-ADP relationship involves implicitly the V’O_2_-P_i_ dependence), and in Table 1. V’O_2_, ADP and P_i_ are considerably higher at rest than in state 4_id_. Δp and ATP/ADP are lower at rest. During moderate and intensive work V’O_2_, ATP/ADP and Δp are much higher than in state 3_id_, while ADP and P_i_ are lower. During intensive work V’O_2_, ADP and P_i_ are higher, while ATP/ADP and Δp are lower than during moderate work. In intermediate state V’O_2_ is much lower than during work. However, the values of ADP, ATP/ADP, P_i_ and Δp in intermediate state are quite similar to that during work. In fact, they are located between the values for moderate work and intensive work.

**Fig 2:**
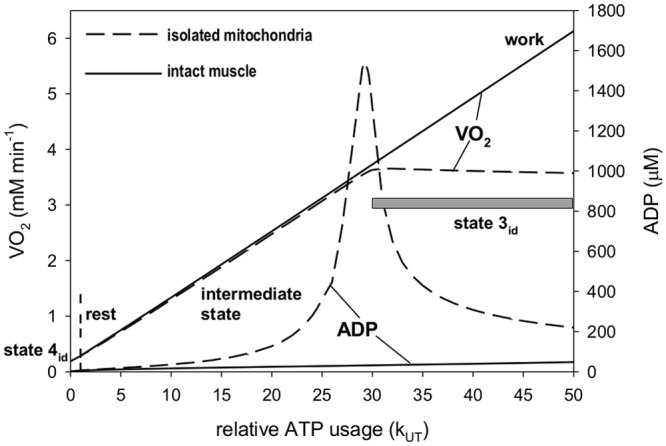
Simulated steady-state dependence of V’O_2_ and ADP on the relative ATP usage activity. The simulations are made for the system without ESA (isolated mitochondria) and with ESA (intact skeletal muscle). ATP usage activity corresponds to the rate constant of ATP usage k_UT_, scaled to 1 in resting muscle. V’O_2_ in isolated mitochondria is scaled for mitochondria in skeletal muscle in order to make a direct comparison.

During state 4_id_-state 3_id_ transition at some value of the activity of ATP usage (k_UT_ rate constant) the OXPHOS capacity for ATP production becomes saturated and V’O_2_ does not increase further with an increase in k_UT_. In the result, a huge increase in ADP takes place, at the cost of a decrease in ATP. This is shown in Fig. 2. When k_UT_ is increased even further, ADP decreases as it is converted into AMP (reaction catalyzed by adenylate kinase, AK). Nothing like this happens during rest-work transition in intact skeletal muscle. V’O_2_ increases linearly with k_UT_. Also only a moderate increase in ADP takes place. This is shown in Fig. 2. OXPHOS does not become saturated when k_UT_ increases, even at intensive work (relative k_UT_ = 80, outside Fig. 2). These differences between intact skeletal muscle and isolated mitochondria result in the completely different phenomenological V’O_2_-ADP relationships presented in Fig. 1.

The distribution of metabolic control over V’O_2_ among OXPHOS, proton leak and ATP usage changes very significantly between different states. This is demonstrated in Table 2. In state 4_id_ about three fourth of the control is kept by proton leak and about one fourth by OXPHOS (by definition ATP usage is absent in state 4_id_). In intermediate state almost all the control is exerted by ATP usage. In state 3_id_ almost all the control is shifted to OXPHOS. In intact skeletal muscle at rest about half of the control is at proton leak (the greatest FCC as in state 4_id_), while one third of the control is at ATP usage (unlike in state 4_id_). At moderate and high work intensity almost all the control is exerted by ATP usage. Therefore, the control pattern during work resembles that in intermediate state and not in state 3_id_.

**Table 2:** Simulated flux control coefficients (FCCs) over V’O_2_ of OXPHOS, proton leak and ATP usage in different states.

State (relative k_UT_)	OXPHOS	proton leak	ATP usage
State 4 _id_ (0)	0.24	0.76	-
Intermediate state (15)	0.02	0.04	0.94
State 3 _id_ (31)	0.94	0.01	0.03
Rest (1)	0.16	0.49	0.35
Moderate work (30)	0.01	0.02	0.96
Intensive work (90)	0.00	0.01	0.99

The activity of ATP usage (k_UT_ rate constant) is scaled for 1 at rest.

**Fig 3:**
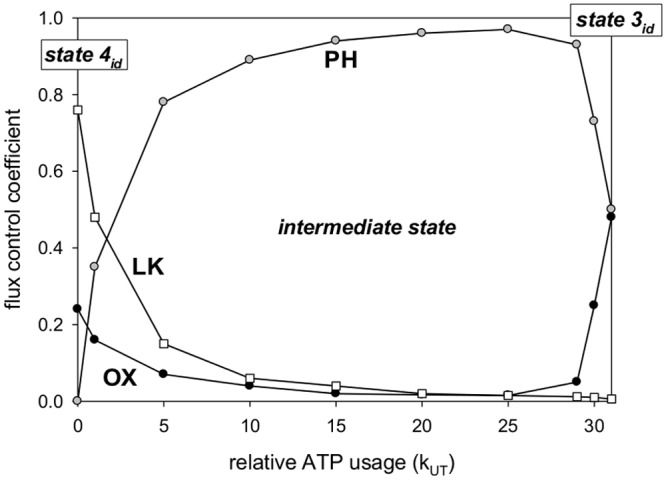
Simulated flux control coefficients (FCCs) over V’O_2_ for different OXPHOS subsystems. The simulations refer to isolated mitochondria. Oxidation (OX), phosphorylation (PH) and proton leak (LK) subsystems were distinguished around protonmotive force (Δp) as within top-down approach [24,4] to Metabolic Control Analysis.

The system can be also divided into blocks in a different way: into oxidation block, phosphorylation block and proton leak, as it was done within the top-down approach to Metabolic Control Analysis around Δp (or ΔΨ) [24,4]. The simulated control over V’O_2_ by oxidative subsystem (OX: NADH supply, complex I, complex III, complex IV), phosphorylation subsystem (PH: ATP synthase, ATP/ADP carrier, P_i_ carrier, ATP usage) and proton leak subsystem in state 4_id_, state 3_id_ and in intermediate states is presented in Fig. 3. Computer simulations predict that LK has the greatest FCC in state 4_id_, PH exerts almost all the control in intermediate state, while PH and OX contribute equally to the control in state 3_id_. This set of simulations was added in order to further test the model by comparing theoretical predictions with experimental data.

## Discussion

In the present theoretical, research-polemic study ‘idealized’ state 4, state 3 and the intermediate (in terms of ATP synthesis, V’O_2_, ADP, ATP/ADP, P_i_ and Δp) state in isolated mitochondria are compared with resting and working states in intact skeletal muscle. Computer simulations confirm the previous experimental observation [8] that the resting state does not correspond exactly to state 4 and, first of all, strongly suggest, again—in agreement with experimental data—that the working state is very different from state 3. It is postulated that the differences between isolated mitochondria (at least in the absence of Ca^2+^) and intact muscle in the mechanisms responsible for the regulation of OXPHOS when ATP demand (rate constant of ATP usage, k_UT_) increases are responsible for the latter difference. Therefore, it seems that isolated mitochondria (at least in the absence of Ca^2+^) are not a good model of the regulation of OXPHOS during work transitions in intact tissues, in particular in intact skeletal muscle.

A great variety of different states 4 and states 3 are present in experimental studies, even in mitochondria isolated from the same cells/tissues. For instance, Brand and Nicholls [7] distinguish state 4 (without oligomycin) and state 4_oligomycin_ (with oligomycin that inhibits ATP production by ATP synthse and therefore prevents any dissipation of the protonmotive force (Δp) even in the presence of external ATPases in mitochondria preparations). The quality of mitochondria preparation, expressed e.g., as a fraction of mitochondria with intact (unbroken) inner membrane, affects strongly V’O_2_ in state 4 and RCR (respiratory control ratio). Disruption of the inner membrane in some fraction of mitochondria elevates state 4 respiration and diminish RCR, because these mitochondria are in fact in state 3_unc_, where the proton leak is maximal and is responsible for essentially whole mitochondrial oxygen consumption. The respiratory substrates used in the isolated mitochondria system also could affect state 4 and RCR. Therefore many different experimental states 4 could be defined, for instance state 4_succ,RCR = 2_ (with succinate and a relatively high fraction of mitochondria with disrupted inner membrane) or state 4_pyr/mal,RCR = 10,oligomycin_ (with pyruvate/malate, low fraction of mitochondria with disrupted inner membrane and oligomycin).

Similarly, many different ‘experimental’ states 3 can be distinguish. ‘State 3’ can be induced by ADP addition, high hexokinase amount/activity or uncoupler addition. Different respiratory substrates are used in experimental studies on isolated mitochondria, for instance succinate, pyruvate/malate, glutamate/malate, fatty acids, or even ascorbate + TMPD or external fully-reduced cyt. c. Different fraction of mitochondria remains intact after different isolation procedures (V’O_2_ in state 3_unc_, similar to the state present in mitochondria with a broken inner membrane, is higher than in state 3 induced by ADP or hexokinase+glucose addition). Therefore, again, different ‘experimental’ states 3 can be defined, for instance state 3_ADP,succ_ (with high ADP and succinate), state 3_hex,pyr/mal_ (with hexokinase + glucose and pyruvate/malate) or state 3_unc,glu/mal_ (with uncoupler and glutamate malate) (compare, for instance, [29]).

Therefore, I suggest to define ‘idealized’ state 4 and state 3 (state 4_id_ and state 3_id_) in relation to the plethora of ‘experimental’ states 4 and states 3. The criteria of state 4_id_ and state 3_id_, quoted in Theoretical Methods (intact inner membrane in 100% of mitochondria, 100% of V’O_2_ in state 4_id_ due to proton leak, ‘physiological’ respiratory substrates, low P_i_ in state 4 and high P_i_ in state 3, no uncoupler in state 3_id_, lack of direct OXPHOS activation) are perhaps somewhat arbitrary and I am open to discussion on how to improve them and possibly to extend their list.

Of course, it is not possible to achieve state 4_id_ and state 3_id_ in 100% in the experimental way. For many purposes trying to approach them would be simply unpractical, contrary to the aim of an experiment or even counter-productive. However, they could constitute a reference point for various ‘experimental’ states 4 and states 3 useful in a general discussion. On the other hand, if somebody would intend to approximate state 4_id_ and state3_id_ in the experimental way, the present study can offer some suggestions how to do this. For instance, computer simulations predict that P_i_ concentration in state 4_id_ would be about 0.5 mM (compare Table 1).

State 4_id_ and state 3_id_ are used in computer simulations in the present study. They reflect most of different ‘experimental’ states 4 and states 3 at least semi-quantitatively. Of course, some aspects of state 4_id_ and state 3_id_, for instance the ‘physiological’ respiratory substrate mixture, are not taken into account explicitly within the computer model used. ‘Idealized’ intermediate state was not defined formally and strictly in Theoretical Methods, as it comprises in fact a continuous spectrum of states, but it can be regarded as a set of states intermediate between state 4_id_ and state 3_id_, with intermediate ATP usage activity.

Computer simulations confirm the experimental findings [8,9] that the resting state differs from state 4 (in particular—from state 4_id_), because in the former there is some ATP usage for basal processes keeping the cell alive (RNA/protein synthesis, Na^+^/K^+^ and Ca^2+^ ion circulation), while there is by definition no ATP usage in the latter. All cell types have common basal ATP-using processes (see e.g., [43]). For this reason, in resting skeletal muscle proton leak accounts for only about 60% of V’O_2_ [8,9], while, again by definition, 100% of V’O_2_ in state 4_id_ is due to proton leak. In the result, not only V’O_2_, but also ADP and P_i_ are higher, while ATP/ADP and Δp are lower at rest than in state 4 (see Fig. 1, Table 1). Therefore, it can be said that rest is located between state 4_id_ and intermediate state or, if somebody prefers, at the lower extreme of intermediate state—compare Fig. 1. Generally, the resting state can be defined within the regulation of OXPHOS by the negative feedback acting through ADP present in isolated mitochondria—the point representing the resting state lies on the curve representing the V’O_2_-ADP relationship for isolated mitochondria: compare Fig. 1.

A completely different situation takes place when one compares the working state in intact skeletal muscle with state 3 (in particular—state 3_id_) in isolated mitochondria. At moderate and intensive work V’O_2_, ATP/ADP and Δp are much higher, while ADP and P_i_ much lower than in state 3. The phenomenological V’O_2_-ADP relationship is much steeper during rest-work transition than during state 4_id_-state 3_id_ transition. This relationship in intact skeletal muscle during rest-intensive work transition encountered in experimental studies is very different in different muscles, but is always much steeper than a first-order dependence (see [36,21] for overview). According to the author’s proposal, this is caused by a fundamental difference in the mechanisms responsible for OXPHOS regulation between intact skeletal muscle and isolated mitochondria in the absence of external Ca^2+^. While in the latter case the only relevant mechanism is the negative feedback acting through ADP (constant or nearly constant P_i_ is fixed), it was postulated that in the former case this mechanism co-operates with the each-step activation mechanism (ESA) [22,20,21]. This is the so-called mixed mechanism (MM), where all OXPHOS complexes are directly activated, but to a smaller extent than ATP usage, and therefore some moderate changes in ADP and other metabolites take place [21]. In the author’s opinion, this differs skeletal muscle from intact heart in vivo, where ‘pure’ ESA operates: all OXPHOS complexes are directly activated to the same extent as ATP usage and the concentrations of intermediate metabolites (ADP, ATP, P_i_, PCr, NADH) remain essentially constant during work transitions [21]. Generally, according to the author’s proposal, ESA or, more precisely, MM causes that the working state in intact skeletal muscle is so different from state 3 in isolated mitochondria.

In fact, in terms of ADP, P_i_, ATP/ADP and Δp moderate and intensive work resemble much more the intermediate state than state 3 (in particular—state 3_id_). This can be seen in Fig. 1 and Table 1. Of course, this does not concern V’O_2_, which is much lower in the intermediate state not only than at moderate and intensive work, but also than in state 3_id_.

In isolated mitochondria (especially in the absence of external Ca^2+^) the OXPHOS capacity for ATP synthesis (and therefore oxygen consumption) becomes saturated at some value of ATP usage activity (relative k_UT_, scaled to 1 in resting skeletal muscle). In the present theoretical study this happens at relative k_UT_ equal to about 31. Increasing k_UT_ above this value does not cause a further increase in V’O_2_ (see Fig. 2). Thus, state 3_id_ is reached. At the same time ADP increases dramatically, at the cost of ATP. When ATP usage activity increases even further (over the relative value of 31), ADP decreases as it is converted to AMP (reaction catalyzed by adenylate kinase).

The situation is completely different in intact skeletal muscle. Here, V’O_2_ increases linearly with energy demand (k_UT_) and only a moderate increase in ADP following the increase in k_UT_ takes place. This is caused, according to the author’s proposal, by ESA mechanism (or, more precisely, MM) present in intact muscle. The work and state 3_id_ V’O_2_ diverge in computer simulations at k_UT_ ≈ 30, but ADP becomes to differ significantly between the isolated mitochondria (intermediate state) and intact muscle (work state) system already above the resting ATP demand (relative k_UT_ = 1) (see Fig. 2).

The simulations presented in Fig. 2 demonstrate also that there is no uniform state 3_id_. Namely, when the relative activity of ATP usage (k_UT_) increases from 31 (beginning of state 3) to 50, V’O_2_ is maintained on a constant ‘saturated’ level, but ADP gradually decreases, as it is converted to AMP (through adenylate kinase, AK, reaction equilibrium). Therefore, the values of ADP, ATP/ADP, P_i_ and Δp given in Table 1 are valid just for one particular activity of ATP usage (relative k_UT_ = 31), at which the OXPHOS capacity for ATP synthesis becomes completely saturated.

Generally, the idea of each-step activation (ESA) and computer simulations based on it (compare Fig. 1, Table 1) lead to the conclusion that the maximum V’O_2_ per mitochondria amount in intact skeletal muscle can be significantly higher than in isolated skeletal muscle mitochondria (this conclusion is not obvious, because the maximum V’O_2_ can be determined by the maximum ATPases activity or O_2_ supply, and not by maximum OXPHOS activity). Tonkonogi and Sahlin [40] estimated on the basis of experimental data that the maximal V’O_2_ in intact human skeletal muscle during voluntary exercise, when recalculated for mitochondria amount, is 2.5–5 times higher than in isolated skeletal muscle mitochondria respiring on pyruvate/malate (NAD-related respiratory substrates). The authors confronted their own (and also made by other authors) measurements of V’O_2_ in isolated mitochondria in state 3, skinned fibers and muscle homogenates with maximal pulmonary V’O_2_ during whole body exercise and single muscle exercise in humans [40]. After recalculation for skeletal muscle cells in vivo, these values would equal 3–4 mM min^-1^ for isolated skeletal muscle mitochondria respiring on pyruvate/malate, about 8 mM min^-1^ in intact skeletal muscle during whole-body exercise and about 16 mM min^-1^ for single-muscle exercise [16]. When the maximal V’O_2_ for single human quadriceps exercise measured by Richardson and co-workers [45] is used for calculations (after recalculation: about 26 mM min^-1^), an about 7–8-fold higher maximal V’O_2_ in intact muscle than in isolated mitochondria would be obtained. These estimations agree very well with the conclusions drawn in the present article: see Fig. 1, Table 1 and the relevant discussion. Schwerzmann and co-workers [46] measured the maximal V‘O_2_ (per mitochondria volume) in isolated skeletal muscle mitochondria incubated with different substrates. They obtained the values of 3.1 ml O_2_ min^-1^ ml^-1^ of mitochondria volume for pyruvate/malate (NAD-related substrates), 5.8 ml min^-1^ ml^-1^ for succinate (FAD-related substrate) and 14.5 ml min^-1^ ml^-1^ for external reduced cytochrome c (cyt. c). The authors concluded that ‘Oxidative activities of 3.1 ml O_2_ min^-1^ ml^-1^ with pyruvate/malate and 14.5 ml min^-1^ ml^-1^ with cytochrome c as substrates were theoretical lower and upper bounds’ and that the value of V’O_2_ for succinate corresponds well to the maximal V’O_2_ in intact skeletal muscle (recalculated for mitochondria content) estimated for up to 5 ml min^-1^ ml^-1^ by Hoppeler and Lindstedt [47]. However, both succinate and, especially, cyt. c are completely unphysiological as main or exclusive respiratory substrates in skeletal muscle and the maximal V’O_2_ for a given maximal ATP production by OXPHOS is much overestimated with these substrates in relation to in vivo conditions. In intact muscles during e.g. carbohydrate oxidation most electrons (e^-^) are transferred from respiratory substrates to the respiratory chain through NAD (5 NADH molecules are produced per 1 pyruvate molecule: by glyceraldehyde-P dehydrogenase, pyruvate dehydrogenase, isocitrate dehydrogenase, 2-oxoglutarate dehydrogenase, malate dehydrogenase), while FAD plays only a minor role (1 FADH_2_ molecule produced per 1 pyruvate molecule: by succinate dehydrogenase). No e^-^ are transferred from outside of the respiratory chain directly on cyt. c. The transfer of 2 e^-^ from NAD to oxygen is coupled with pumping of 10 e^-^ through the inner mitochondrial membrane (complex I, III and IV), transfer from FAD: with pumping of 6 e^-^ (complex III and IV) and transfer from cyt. c: with pumping of 2 e- (complex IV). Therefore, the O_2_/ATP ratio for pyruvate/malate will be 10/6 = 1.67 times smaller than for succinate and 10/2 = 5 times smaller than for cyt. c (in fact, the last value is somewhat overestimated, because cytochrome oxidase transfers two protons, but four charges). Consequently, the V’O_2_ for a given maximal rate of ATP production would be appropriately greater for succinate and cyt. c than for pyruvate/malate. This agrees well with the results obtained by Schwerzmann and colleagues who obtained 5.8/3.1 = 1.86 times higher maximal V’O_2_ with succinate than with pyruvate/malate and 14.5/3.1 = 4.68 times higher maximal V’O_2_ with cyt. c than with pyruvate/malate. Additionally, pyruvate carrier and complex I that have a significant control over V’O_2_ in state 3 [48,37] are ‘omitted’ with succinate as substrate (pyruvate carrier is also ‘omitted’ with glutamate/malate), while most OXPHOS complexes that keep almost entire control over V’O_2_ [48,37] are ‘omitted’ with cyt. c (or ascorbate + TMPD) as substrate. Finally, cyt. c in vivo is reduced in 20–30% and not in 100%. However, as discussed above, pyruvate/malate are decidedly more physiologically relevant respiratory substrates than succinate or cyt. c. For this reason the value of the maximal V’O_2_ of 3.1 ml min^-1^ ml^-1^ for isolated mitochondria respiring on pyruvate/malate should be compared with the value of about 5–8 ml min^-1^ ml^-1^ for single-muscle exercise in intact skeletal muscle [44,45]. Therefore, the final conclusions would be similar to that drawn by Tonkonogi and Sahlin [40]: that the maximal V’O_2_ in intact skeletal muscle is much higher than in isolated mitochondria. Finally, one should be aware of the fact that isolated mitochondria in state 3 are saturated with ADP, which does not take place in the case of mitochondria in situ. This further increases the discrepancy between isolated mitochondria and intact skeletal muscle. Therefore, the highest V’O_2_ in intact muscle under physiological conditions is not really ‘maximum’. And, to repeat this statement once again, the presence of ESA does not necessarily imply that the maximum V’O_2_ in intact muscle is higher than in isolated mitochondria in the case where the former is determined by maximum ATPases activity or oxygen supply, and not by maximum OXPHOS activity. Therefore, the difference in the phenomenological slope of the V’O_2_-ADP relationship between intact muscle and isolated mitochondria is more important than the difference in the maximum V’O_2_, although the discussed experimental data clearly show that the latter is a fact.

In different muscles/experimental conditions very different (slopes of) phenomenological V’O_2_-ADP relationships during rest-to-work transitions are encountered in experimental studies (see [21] for overview). These differences can be easily explained by different values of the power coefficient x in the ‘n^x^’ kinetic description of ESA (see Theoretical procedures) corresponding to different ESA intensities and giving a family of phenomenological V’O_2_-ADP relationships with different slopes [21]. Therefore, the simulated phenomenological V’O_2_-ADP relationship for intact muscle shown in Fig. 1 is just an example (in fact, it represents a very moderate ESA intensity: compare Figures Five and Nine in [21])—curves with very different slopes would have to be fitted, by manipulation with x value, to different experimental results.

In fact, different work states can be distinguished and named, similarly as in the case of ‘experimental’ states 4 and states 3. They could be characterized, for instance, by the relative activity of ATP usage in relation to rest (n) and the intensity of ESA coefficient x. For example, the intensive work simulated in the present study, where n = 80 and x = 0.35, can be described as work_80,0.35_.

The pattern of metabolic control over V’O_2_, expressed as the values of flux control coefficients (FCCs) for particular elements of the system, changes very significantly between different states in isolated mitochondria and intact skeletal muscle. This is demonstrated in Table 2 (compare also computer simulations in [38]). In state 4_id_ most of the control is exerted by proton leak, the rest being at OXPHOS complexes. In this state there is by definition no ATP usage and therefore FCC of this process is irrelevant for this state. The situation becomes dramatically different in the intermediate state, where almost all the control is kept by ATP usage. In state 3_id_ a subsequent dramatic change in control pattern takes place, because essentially entire control is taken over by OXPHOS complexes. The distribution of the control among OXPHOS complexes is more or less uniform: compare computer simulations in [38,21]. This prediction agrees very well with experimental data [3,48,37] (in fact the computer model used in the present study was developed to take into account the uniform control distribution [38]).

In intact skeletal muscle at rest proton leak has the greatest FCC, but smaller than in state 4_id_ (Table 2). The rest of the control is exerted by ATP usage (unlike in state 4_id_) and OXPHOS (like in state 4_id_), as encountered in experimental studies [8]. During moderate and intensive work almost all of the control is at ATP usage. This is because OXPHOS does not become saturated with ATP usage intensity (ADP concentration is still relatively low) at higher k_UT_ values (compare Fig. 2). As discussed above, this is due to the presence of ESA (or, more precisely, MM). The simulated pattern of metabolic control over V’O_2_ in different states demonstrates that moderate and intensive work resemble much more intermediate state than state 3_id_ not only in terms of ADP, ATP/ADP, P_i_ and Δp, but also in terms of FCCs.

Generally, the simulated pattern of metabolic control in different states obtained in the present study agrees well, at least semi-quantitatively, with that measured in experimental studies [3,48,37,4,8].

Computer simulations concerning the distribution of control among oxidative subsystem (OX: NADH supply, complex I, complex III, complex IV), phosphorylation subsystem (PH: ATP synthase, ATP/ADP carrier, P_i_ carrier, ATP usage) and proton leak subsystem (LK) in state 4_id_, state 3_id_ and intermediate states (Fig. 3) agree well with FCCs for these subsystem measured in the experimental way in isolated mitochondria [4] (however, one should bear in mind that the authors used liver mitochondria respiring on succinate). LK has the greatest control in state 4/state 4_id_, PH exerts almost entire control in intermediate state, while PH and OX contribute equally to the control in state 3/state 3_id_. However, within the PH subsystem, ATP usage predominates as the controlling step in intermediate state, while in state 3/state 3_id_ its FCC is close to zero and the control is taken over by the remaining elements of this subsystem (ATP synthase, ATP/ADP carrier, P_i_ carrier) (compare Table 2, Fig. 3 and [3,38]). In intact muscle, LK exerts about 50% of the control at rest, while almost entire control is taken over by the PH subsystem (almost exclusively ATP usage) during work.

The computer model of the skeletal muscle bioenergetic system used in the present study certainly contains several approximations and simplifications. For instance, it does not contain a detailed kinetic description of TCA cycle, glycolysis or metabolite exchange with blood, as some other models do (compare e.g., [17,49,50]). However, these aspects of the system are not directly related to the topic of the present article.

## Conclusions

The present theoretical, research-polemic study demonstrates that it is possible to unify the kinetic behavior of OXPHOS (oxidative phosphorylation) in isolated mitochondria and intact skeletal muscle during varying energy (ATP) demand using a unique kinetic description of the system, under assumption that the ESA (each-step activation) mechanism is present in intact muscle at work. It confirms earlier experimental findings that resting intact skeletal muscle is not exactly in state 4 and, first of all, it strongly suggests that the working state in intact muscle is very different from state 3 in isolated mitochondria. ‘Idealized’ state 4 and state 3 (state 4_id_ and state 3_id_) that are intended to serve as a reference for various ‘experimental’ states 4 and state 3 are defined. Computer simulations show that V’O_2_, ATP/ADP and Δp are much higher, while ADP and P_i_ much lower at work in skeletal muscle than in state 3_id_ in mitochondria. The phenomenological V’O_2_-ADP relationship during rest-work transition is much steeper than during the state 4_id_-state 3_id_ transition. It is postulated that the huge differences between intact muscle and isolated mitochondria are caused by the presence of the each-step activation (ESA) mechanism in intact skeletal muscle, which is absent in isolated mitochondria (at least in the absence of Ca^2+^). The metabolic control over V’O_2_, characterized by flux control coefficients (FCCs), is dominated by proton leak in state 4 and, to a smaller extent, in the rest state. Almost all of the control is kept by OXPHOS complexes in state 3. During moderate and intensive work in intact skeletal muscle as well as in intermediate state in isolated mitochondria (a state intermediate between state 4 and state 3) ATP usage is the main controlling process. Generally, the working state in muscle resembles much more intermediate state than state 3_id_ in isolated mitochondria in terms of ADP, P_i_, ATP/ADP, Δp and FCCs, but not in terms of V’O_2_. The present study suggests that isolated mitochondria (especially in the absence of Ca^2+^) cannot serve as a good model of OXPHOS regulation and bioenergetic system behavior in intact skeletal muscle. It also shows that the computer model used for simulations and the postulated each-step activation mechanism are able to integrate and explain the (differences in the) kinetic behavior of the energy metabolism in intact skeletal muscle and isolated muscle mitochondria in response to elevated energy demand.
